# Supplementary material for: Prevalence of heated tobacco product use among adolescents in Taiwan
Source: PLoS One. 2020 Dec 21;15(12):e0244218. doi: 10.1371/journal.pone.0244218 (PMC7751857; doi:10.1371/journal.pone.0244218)
Supplement: Attachment — Submitted filename: Response to Reviewers-0901-OK.docx [file pone.0244218.s001.docx]

Review Comments to the Author

Reviewer #1: I found that the paper, titled “Prevalence of Heated Tobacco Product Use among adolescents in Taiwan”, was interesting to read. Because although HTP products were not sold legally in Taiwan, there were the students who had already experienced in using IQOS and who were currently using it. The findings of this paper can be useful, but I suppose the authors should revise and improve the manuscript based on the comments below.

Major comments

I think the manuscript is too brief and has missed some of the useful information for the readers. I suggest that the authors should explain in detail how the students can buy or get HTP products in the Taiwanese tobacco market where does not allow HTP use and also provide more detailed information about HTP black market in Taiwan.

**Response:** To the Introduction, we have added further details pertaining to the HTP market in Taiwan. Despite the fact that HTP products are not sold legally in Taiwan, many young people still have access to them via the internet (e.g., Facebook groups, LINE groups, etc.). There are also a few large-scale e-cigarette or IQOS stores on the market that operate brick-and mortar stores.

In the discussion section, the authors compared their findings with the Korean case. The comparison was only focusing on the prevalence of HTPs among the Korean youth and the Taiwanese youth. It is better to discuss the difference between the Korean HTP market and the Taiwanese HTP market.

**Response:** We have detailed the difference between the Korean and Taiwanese HTP markets in the Discussion section.

Our usage statistics in the current study are slightly lower than those in a report on young Korean adults by Kim et al., in which 5.7% of the study population had previously tried IQOS and 3.5% were current users. The differences can be attributed largely to the study methods (school-based survey vs. online survey), the size of the sample, and the age of the respondents (12–18 years vs. 19–24 years). Nonetheless, we believe that the main reason for the discrepancy is a difference in attitudes toward IQOS use. IQOS products were introduced in Korea in 2017, and the devices are currently available in convenience stores. By contrast, Taiwan forbids the sale of IQOS, with the result that people must resort to online sales or illegal purchases. It should be noted that despite these impediments to accessing the product, the difference in usage behavior between the two countries is really very small, indicating that this issue requires attention.

The authors mentioned several research limitations, but they did not discuss how they had overcome them.

**Response:** We have rewritten the discussion of limitations in the Discussion section.

This study was limited by a number of issues. First, the cross-sectional data in this study was limited to factors associated with the use of IQOS; therefore, we were unable to determine whether the study subjects had used cigarettes or e-cigarettes before using IQOS (i.e., potential causality). Thus, we were able to report only on the correlations among IQOS use, cigarette use, and e-cigarette use. In the future, researchers could conduct follow-up studies to identify the issues underlying IQOS use. Second, the fact that the survey was self-administered means that the actual smoking behavior of the subjects could not be verified, which may have compromised estimates pertaining to the prevalence of IQOS use. Note that we deleted inconsistent answers to tobacco-related questions from the questionnaire. Finally, this survey did not ask the participants how they purchase or otherwise obtain HTP products. In the future, researchers could focus on the specific types of HTP being used by young people as well as information pertaining to pricing and availability.

There was no information regarding IRB.

**Response:** This study used secondary data provided by the Health Promotion Administration of Taiwan, and ethical approval for the current study was granted by the Institutional Review Board of National Yang-Ming University (YM108025E). As for the conduct of the Taiwan **Global Youth Tobacco Survey,** it is an official investigation implemented annually by the Health Promotion Administration, which has also passed the IRB review (EC1041011-F-R2). We have included this information in the Methods section.

Minor comments

Abstract

Cigarettes can be revised to conventional cigarettes.

Methods

It will be better to add a figure regarding the sampling selection process.

More information about the GYTS study need to be added.

Results

95% CI should be added after OR

**Response:** We have modified the manuscript in accordance with the above comments.

Reviewer#2: This paper describes a study aimed at assessing the awareness and use

of the IQOS heated tobacco product system by Taiwanese youth. As PMI seeks to expand IQOS to new markets around the world and has recently garnered US FDA authorization to market IQOS in the USA with a reduced exposure statement, the current study is timely as regulators, policy makers, and others seek independent data on the impact of this product on youth tobacco use. The findings of this study, notably 2% were currently using and 4% had used, are quite surprising given that IQOS was not legally marketed in Taiwan. Other studies have found IQOS use by youth but not to my knowledge this much use in a country where it is not legal to market IQOS. This is a noteworthy finding that would likely be of much interest and concern to tobacco control folks. The data source is a large sample of students from the Taiwan (2018) GYTS and is appropriate for the research questions of interest. The primary limitation of this study is that it does not explain how the substantial number of Taiwanese youth IQOS users are obtaining IQOS and for current users, their supply of Heatsticks (HEETS), when the product is not legally marketed there. If there are GYTS data that could illuminate this, I would encourage their inclusion in the paper. In the absence of such data, the authors could perhaps note this as a limitation and offer informed hypotheses for future research in the discussion section. Additional, generally minor, comments and suggestions are provided below:

**Response:** The GYTS does not inquire about how the participants obtain HTPs. Nonetheless, in the Introduction section, we have added a description of how young people usually obtain HTPs. In the Limitations section, we also recommend that researchers focus on the specific types of HTP being used by young people as well as information pertaining to pricing and availability.

1. Abstract and introduction (1st paragraph): It has been claimed that IQOS is or was an acronym for “I-Quit_Ordinary-Smoking” but PMI folks have denied this and I’ve seen no evidence. Unless the authors have evidence of this meaning of IQOS that they can cite, I suggest removing it.

**Response:** We have removed the words mentioned above.

2. Introduction, 1st paragraph: This is a narrow definition of heated tobacco products. Some HTPs do not use sticks (PAX, Ploom Tech).

**Response:** We have modified the definition of HTPs.

3. Introduction, 2nd paragraph: Please note the nationality and year for these studies. Kim et al. was of a S. Korean population. It is also worth noting for this study that it was an online sample and not designed to yield population estimates. For the Czoli et al. study, please provide year. It may also be worth noting that when this study was conducted (2017), IQOS was not legally marketed in the US (like Taiwan in the present study).

**Response:** We have revised the description in the second paragraph of the Introduction section.

4. Introduction, 3rd paragraph: This section could be much stronger and more relevant. For instances, a link between e-cigarette use and current allergic rhinitis is not directly relevant to the current study nor is the sentence on EVALI. A brief summary of the literature on the toxicity and health effects of IQOS would be fine to highlight the significance of this research, but as the health effects of IQOS are not the subject of this study, the health effects shouldn’t be belabored. Rather, the manuscript space could be used to provide better coverage of the research literature that is more relevant. For instance, there is a study that examined youth use/awareness in Italy and reports documenting the youth appealing marketing of IQOS (for example, see Reuters investigative report on IQOS use of young social media influencers and advertising captured by the Campaign for Tobacco Free Kids).

**Response:** We have removed this paragraph and added new articles pertaining to the use of IQOS and HTPs in other countries.

5. Introduction, final paragraph (p. 6): Please provide a citation for and discuss the study that found that “the prevalence of IQOS use by young people is far from negligible.” If referring to the present study, then please remove the sentence, as it is not common to report the findings/conclusions in the introduction section.

**Response:** We have revised this paragraph in the Introduction section.

6. p. 8 (“After sample weighting,… using an IQOS product”). First, it is atypical to provide results in the method section. But more critically, these estimates of IQOS use do not make sense as the frequencies are more than one-half the size of the 15-24 year-old Taiwanese population. The sentence is also grammatically awkward (“had answering”) and estimates are not accompanied by 95% confidence intervals.

**Response:** The above-mentioned sentences describe the weighted sample size of the two dependent variables. The estimates of IQOS use can be explained by the difference in the number of respondents who provided answers pertaining to the two dependent variables. We have revised the sentences to clarify this issue and added a figure (Fig. 1) illustrating the sample selection process.

After sample weighting, the number of students who responded to the ever use IQOS item (“Have you ever used an IQOS?”) was equivalent to 1,498,502 in the general population. Likewise, the number of students who responded to the current use IQOS item (“During the past 30 days, have you used an IQOS?”) was equivalent to 1,501,527 students in the general population.

7. P. 8, Variables (1st paragraph): Was a picture and/or description of IQOS provided? In US e-cigarette research with youth, it has been commonly observed that providing a description with pictures improves measurement of awareness and use. If a description/picture were not provided, how do we know that some youth are not confusing IQOS for something else?

**Response:** No images of the IQOS device were included in the research questionnaire; however, IQOS was indicated as a heated tobacco product. Note also that electronic cigarettes and IQOS were discussed using separate questions.

8. P. 9 (“The smoking status among close friends was categorized as… no choice”) – it is not clear what this means.

9. P. 11 (“A comparatively high percentage…”): This sentence is nearly identical to the prior sentence.

10. P. 12 (“Low/moderate allowance was inversely correlated with the likelihood of previously trying IQOS”): It is hard to interpret the direction of this finding and the two odds ratios provided are not clear. In general, for clearer communication of the results, I suggest a structure along the lines of: “Having X was associated with an X times greater (or lower) odds of Y.”

**Response:** We have revised the manuscript in accordance with the above comments.

11. Discussion (“The usage statistics… similar report on young Korean adults by Kim et al.”): I would not compare the results of this study with the Kim et al. study. Besides the different age population, the Kim et al. study was of a relatively small, online nonprobability sample. The amount of youth use in this study is surprising given it isn’t legally marketed in Taiwan and greater use in Taiwan than in S. Korea would be similarly surprising because it is legally marketed there. But, estimates are simply not comparable between the two studies.

**Response:** We agree that these two studies cannot be compared. As far as the utilization rate is concerned, we have revised the paragraph in accordance with the opinion of the first reviewer.

12. In Table 1, what was striking was that among those who previously tried IQOS, 78% were not past month smokers and 88% were not past month e-cigarette users. So, while smoking is predictive of IQOS use, the majority of prior (and current) IQOS users were not current/recent smokers or e-cigarette users. This warrants closer examination and discussion as it has potential implications for the population health impact of IQOS.

**Response:** We have changed Table 1 and Table 2 to have the percentages listed in columns.

We have determined that the use of conventional cigarettes and the use of e-cigarettes are both important factors pertaining to the use of IQOS. In other words, current users of cigarettes or e-cigarettes were more likely to have previously used or be currently using IQOS.

We also established a second model in which the independent variables were combined as a single variable indicating smoking in general, which was then divided into the following four categories: cigarette use only, e-cigarette use only, dual-use, and none.

We have revised the manuscript in the Methods, Results, and Discussion sections to clarify this issue.
