# Supplementary material for: Prevalence of heated tobacco product use among adolescents in Taiwan
Source: PLoS One. 2020 Dec 21;15(12):e0244218. doi: 10.1371/journal.pone.0244218 (PMC7751857; doi:10.1371/journal.pone.0244218)
Supplement: Attachment — Submitted filename: Response to Reviewers-1204-OK.doc [file pone.0244218.s002.doc]

**Review Comments to the Author**

Reviewer #1: I have several minor questions and comments. I hope that the authors add their answers to the manuscript.
Introduction
- HTPs usually stands for heated tobacco products. 'Heat-not-burn tobacco product' is the term that the tobacco industry wanted to call it.
**Response:** We have revised the HTP term to heated tobacco products in the manuscript.
- Why does HTP use ban in Taiwan?
**Response:** Currently, Taiwan is not importing HTPs. (i.e., HTPs are illegal). According to Article 14 of the Tobacco Hazards Prevention Act, “No person shall manufacture, import, or sell candies, snacks, toys, or any other objects in form of tobacco products.” HTPs that contain nicotine are treated as a drug (i.e., a violation of the Pharmaceutical Affairs Act). The Tobacco Hazards Prevention Act was passed in 2009, and since that time, no new regulations pertaining to new tobacco products (e.g., e-cigarettes, HTPs) have been passed. In view of rapid increases in the use of new type tobacco products, amendments to the Tobacco Hazards Prevention Act are urgently required.

- Please provide the year when and where PMI launched IQOS first for the journal readers.
**Response:** Philip Morris International (PMI) introduced the IQOS heated tobacco product in Japan, in November 2014. [1]
- How much is the IQOS device on the internet? Can the Taiwanese kids afford it? How was it marketed on the internet? Do you have any samples of it?
**Response:** We searched Taiwanese websites for IQOS products. Generally, IQOS devices are sold on the Internet for 2500-5500 NTD (about 86.8-191.0 USD, where 1 USD = 28.8 NTD), whereas the Cigarette Bomb costs 1000-2100 NTD (34.7-72.9 USD). We determined in this study that only 16% of adolescents received more than 100 USD in pocket money per month, which means that most of them would be unable to afford an IQOS device. Those that can afford the devices can browse pictures and descriptions of various HTP products and make purchases online. After placing an IQOS product in the shopping cart, the purchaser contacts the seller by joining a LINE group in order to arrange shipment. We do not have a sample of the IQOS device. The above description has not been included in the article because we did not want to provide detailed instructions as to the purchasing process.

- Is there only IQOS available? What about glo from BAT, lil from KT&G or many other devices manufactured by China?
**Response:** A survey of relevant Taiwanese websites revealed that IQOS devices were widely sold IQOS, whereas GLO and LIL were more difficult to find.
- If there are other products apart from IQOS, do you have any reason why you asked only IQOS use?
**Response:** The fact that IQOS is widely sold online implies that it is accessible to many adolescents. Furthermore, the survey was limited to questions pertaining to IQOS use.
Discussion
- Price difference can be considered when you compared to Korea and Taiwan.
**Response:** We have addressed this point in the Discussion section. It is certainly possible that the price difference may account for some of the differences in adoption. South Korea has legalized the use of IQOS; therefore, the price is lower. These products are still illegal in Taiwan; therefore, the price is higher.
Conclusion
- The conclusion of this study is recommending to revise the Taiwanese Tobacco Act. Do you have any insight to provide international readers based on your experience in Taiwan?

**Response:** The sale of HTPs is not legally sanctioned in Taiwan; however, the sale of IQOS from other countries has made it accessible to young people in Taiwan. We believe that governments should decide as soon as possible whether to permit or ban these devices; otherwise, young people will become habituated to purchasing HTPs illegally.

Reviewer #2: The authors were responsive to my comments from the initial review and the manuscript is improved. Remaining issues are relatively minor but if addressed would improve this manuscript, mostly for clarity but also in substance.
1. In response to my initial comments, the introduction was revised to remove less relevant material. This is an improvement. However, while containing some additional helpful elaboration of the research, it does not address the existing research on the youth oriented marketing of IQOS. It might also be worth noting the countries in Asia where IQOS is legally sold. Could these be the sources for IQOS black markets in Taiwan? If not, where?

**Response:** IQOS is legally sold in Asian countries such as Japan and South Korea [2]. At present, those countries are the primary source of black market IQOS products in Taiwan. Most IQOS sold online are listed as a Japanese product.

2. Figure 1 was not found in the manuscript.

**Response:** In accordance with formatting requirements, Figure 1 has been removed from the revised manuscript; however, it is available as a supplementary file.

3. The results, including Tables, are clearer with respect to the direction of effects. However, some text remains less clear, particularly without reference to the Table. Specifically, "The following factors were negatively correlated with the likelihood of previously trying IQOS: low (USD <= 50) and moderate (USD 50-100)...". This is confusing in two aspects. First, this could be misread as higher allowance being associated with lower odds of previously trying IQOS. The authors specify "low (USD <=50) but this is not a standard or clear way of reporting such results. Perhaps more of an issue is that this implies a linear relationship with the relationship as reported in the Table seems to be nonlinear: those with the lowest allowance ($0) were no less likely to use IQOS than those with the highest allowance. Also confusing is that the label for the "low" allowance is USD < 50 even while their is a lower a group with $0. In other words, less than USD50 is not exclusive to USD0.

**Response:** We have revised the results to clarify this point. We observed a nonlinear relationship between monthly allowance and previous use of IQOS. Students with low (USD≦50) or moderate (USD 50-100) monthly allowance were less likely to have tried IQOS than were students with high (USD≧100) monthly allowance. We observed no difference between students with no monthly allowance (USD 0) and those with a high (USD≧100) monthly allowance in terms of previously trying IQOS.

4. Discussion - "Despite the fact that Taiwan does not allow the sale of IQOS, the usage rates were higher than in the US in 2019, the year in which year the US Food and Drug Administration authorized the marketing of IQOS HTPs.": The article referenced is by Dai et al, which used 2019 NYTS data to characterize youth awareness and use of HTP. Dai et al. did not examine IQOS use. And while IQOS was introduced to the US in 2019, it was late September and only in one market (and only two markets through the end of the year). The 2019 NYTS data were collected in early 2019, prior to the introduction of IQOS to the US market. I would suggest not contrasting the findings of the present study with Dai et al.

**Response:** Thank you for reminding us about the time at which 2019 NYTS data was collected and the time that IQOS was authorized for sale in the US. Dai [3] reported that IQOS was only authorized for sale in the US during the survey period (February 15, 2019 – May 24, 2019) and that IQOS was not launched in the US until October 2019. Furthermore, Dai examined the ever use and current use of HTPs, including iQOS, glo, and Eclipse. We have updated the discussion in the revised manuscript.

The usage statistics in the current study are slightly higher than those in Dai’s 2019 report on US students, in which 2.4% of the study population had tried HTPs (including iQOS, glo, and Eclipse) and 1.6% were currently using HTPs. At the time of that study, the sale of IQOS was not sanctioned in Taiwan or the US; however, the usage rates were higher in Taiwan than in the US. Clearly, the use of IQOS products by young people in Taiwan is far from negligible.
5. Lastly - while there are few grammatical or typographical errors, the phrasing of several sentences reads a bit awkward or nonstandard in places. For instance, the sentence ending the first paragraph in the Discussion "use of both cigarettes and e-cigarettes > use of e-cigarette only > use of cigarettes only > none" is a nonstandard (for a Discussion section) use of equality signs (typically would be spelled out). The titles for Tables 3 and 4 are also not standard. For Table 3, for example, consider something more standard such as: Logistic Regression Models for Prior Use of IQOS among Taiwanese Youth (2018). The authors need to use this title; rather, I offer it as an example of a more standard and arguably more informative title. Tables should stand be interpretable separate from the text. These and similar writing issues are more stylistic and minor but if addressed would lead to a more polished paper.

**Response:** We have revised the Discussion section and Tables in the manuscript.

**References**

1.	Tabuchi T, Kiyohara K, Hoshino T, Bekki K, Inaba Y, Kunugita N. Awareness and use of electronic cigarettes and heat-not-burn tobacco products in Japan. Addiction. 2016;111(4):706-13. Epub 2015/11/15. doi: 10.1111/add.13231. PubMed PMID: 26566956.

2.	Glantz SA. Heated tobacco products: the example of IQOS. Tob Control. 2018;27(Suppl 1):s1-s6. Epub 2018/10/26. doi: 10.1136/tobaccocontrol-2018-054601. PubMed PMID: 30352841; PubMed Central PMCID: PMCPMC6252052.

3.	Dai H. Heated tobacco product use and associated factors among U.S. youth, 2019. Drug Alcohol Depend. 2020;214:108150. Epub 2020/07/10. doi: 10.1016/j.drugalcdep.2020.108150. PubMed PMID: 32645682.
